# Ankle Arthrodesis using Ilizarov Ring Fixator: A Primary or Salvage Procedure? An Analysis of Twenty Cases

**DOI:** 10.5704/MOJ.1811.006

**Published:** 2018-11

**Authors:** O Hasan, S Fahad, S Sattar, M Umer, H Rashid

**Affiliations:** Section of Orthopaedics, Aga Khan University Hospital, Karachi, Pakistan

**Keywords:** ankle, arthrodesis, Ilizarov fixator, union, limb salvage

## Abstract

**Introduction:** Ankle arthrodesis using the Ilizarov technique provides high union rate with the added benefits of early weight-bearing, and the unique advantage of its ability to promote regeneration of soft tissue around the bone, including skin, muscle and neuro-vascular structures, and its versatility to allow correction of the position of the foot by adjusting the frame post-operatively as needed. We describe our experience with this technique and the functional outcomes in our patients.

**Materials and Methods:** This retrospective study was conducted in 20 ankle fusion cases using the Ilizarov method between the years 2007 and 2017. We defined success in treatment by loss of preoperative symptoms and radiological union on plain radiographs of the ankle.

**Results:** Fusion was achieved in all patients (100%). Immediate post-operative ambulation was with full weight bearing (FWB) in 16 (83%) of the participants and non-weight bearing (NWB) in 3 patients (17%). Post-procedure 11 patients (67%) of the participants who were full weight bearing required some form of support for walking for 2-3 weeks. Post-operatively three patients had pin tract infection requiring intravenous antibiotics. Radiological union took range of 6-12 weeks, mean union time was 8 weeks. Only one patient required bone grafting due to bone loss. Average follow-up period was 10-45 months.

**Conclusion:** The Ilizarov technique has a high union rate and leads to general favourable clinical outcome and may be considered for any ankle arthrodesis but is especially useful in complex cases such as for revisions, soft-tissue compromise, infection and in patients with risk for non-union. Early weight bearing is an extra benefit.

## Introduction

Ankle arthrodesis is the fusion of the ankle (tibio-talar) joint and is indicated in conditions such as advanced ankle arthritis, post traumatic arthritis, congenital and neuromuscular disorders, infection, avascular necrosis of the talus, advanced posterior tibial tendon dysfunction and Charcot neuroarthropathy, and serves as a salvage procedure for failed total ankle arthroplasty^1^. Arthrodesis is often a limb salvage procedure and an alternative to below knee amputation in patients with end-stage ankle arthritis^2^.

The techniques recorded in literature for arthrodesis up until now include crossed screw structure (two screws inserted from the distal tibia, across to each other, into the talus), intramedullary nail, plate, external fixation frame and so forth; however, there remains much controversy with respect to the optimal technique for ankle fusion to acquire steady rigid fixation, fixation methods that does not allow interfragmentary movement under functional weight bearing and utilizes the compression principle, accompanying restoration of plantigrade foot function^3-6^. Furthermore, existing techniques are associated with several complications such as malalignment, infection, nonunion, and adjacent joint osteoarthritis^7^.

Internal fixation for ankle arthrodesis is sufficient in most cases; however, several types of infections i.e. chronic osteomyelitis and tuberculosis infections, ankle deformity or limb length discrepancy, compromised soft tissue around the ankle and deficient bone stock as well as neurological conditions can result in less than optimal situations for internal fixation. In such conditions Ilizarov fixator is preferred^7-9^. Ilizarov fixator is a versatile device; it provides circumferential rigid fixation and at the same time provides dynamic axial compression, allowing the surgeon to address any intraoperative error or loss of position in early postoperative time^10^. It is a minimally invasive, secure and

successful method for treating these difficult cases of ankle arthrodesis and allows immediate weight bearing^11^. The Ilizarov technique has a high union rate and leads to general improvement in clinical outcome and may be considered as a primary and definitive procedure when expertise is available.

The aim of the present study was to assess the results of the Ilizarov external fixator in performing ankle fusion in 20 ankles. The Ilizarov fixator was applied in patients with severe soft tissue compromise and bone loss, patients with Charcot arthropathy and unstable ankles. We describe our experience with this technique, including all functional and radiological outcomes in our tertiary care university hospital.

## Materials and Methods

This is a study conducted in our university hospital which is a tertiary-care level-1 trauma centre. We obtained the hospital ethical review committee approval and registered the study in data registry.

A retrospective analysis of 19 patients (20 ankles) who underwent ankle arthrodesis with Ilizarov external fixator during the period from September 2004 to May 2017 was conducted. All orthopaedic patients who underwent ankle arthrodesis with Ilizarov external fixator were enrolled into the study. Patients with missing records and those who were lost to follow-up were excluded. Data collected included: age, gender, mechanism of injury, site of impact , type of fracture, history of previous fixation, indication for ankle arthrodesis, whether there was a need for bone grafting, the mean operative time.

Pre-operative assessment included a thorough history, physical examination which included gait analysis, ankle, hind foot range of motion, limb length discrepancy and the current condition of the patient’s relevant soft tissue. Comparison was done with the contra lateral limb. Medical co-morbidities were recorded and controllable risk factors identified and optimised before surgery.

Radiographic evaluation included radiographs of the foot and ankle for preoperative assessment and surgical planning. A magnetic resonance imaging (MRI) was done in selected cases of nonunion to identify the severity and extent of infection and osteomyelitis. In patients with previous history of nonunion septic joint, relevant infection markers like erythrocyte sedimentation rate and C-reactive protein were regularly sampled. All patients were operated on by two senior orthopaedic surgeons with experience in trauma management and with the Ilizarov apparatus. Descriptive statistics mean was calculated for quantitative variables like age of the patients and length of hospital stay, whereas frequency and percentage were calculated for categorical data.

**Fig. 1:**
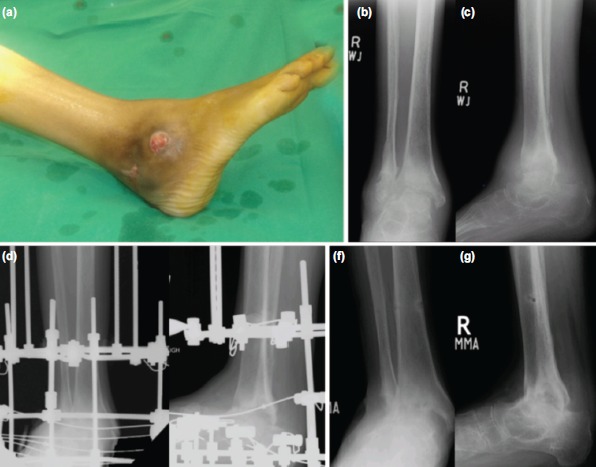
(a) Intra-operative photograph of 45 year-old lady with tuberculosis ankle joint showing ulcers at ankle. (b,c) Pre-operative radiographs Ilizarov anteroposterior and lateral views showing subchondral erosions and sclerosis. (d,e) Immediate postoperative radiographs showing Ilizarov fixator across ankle joint Ilizarov anteroposterior and lateral views. (f,g) Nine months post-operative radiographs Ilizarov anteroposterior and lateral views after removal of Ilizarov fixator showing ankle joint fusion.

All procedures were performed under general anaesthesia. The patient supine on the operating table with elevation under the ipsilateral buttock. Intravenous prophylactic antibiotics were administered at the time of induction of anaesthesia as according to our institution’s guidelines. The ankle joint was approached anterolaterally. Any ulcer over the lateral aspect was excised *en bloc* with the skin incision. Existing implants from previous surgery were removed. In each case a beveled osteotomy was created, from superolateral to inferomedial 3 to 5cm proximal to the level of the ankle joint, taking several centimeters of the distal shaft that was used as autogenous graft if needed. In-situ fusion was performed in the absence of varus/valgus deformity. The dense fibrous tissues and synovium were excised. The articular surfaces of the distal tibia and the dome of the talus were excised with osteotome to allow good co-optation between the distal tibia and the talus with talus aligned 90 degrees to the tibia. The talus was opposed to the distal tibia and held by Kirschner wires inserted from the plantar aspect of the calcaneus to the tibia. Infected cases were addressed through aggressive surgical debridement: bone and soft tissue cultures were sent and an Ilizarov frame application in same sitting (Fig. 1). The Ilizarov frame with two rings for the distal tibia appropriately sized to the leg and a foot plate or mid-foot ring. Compression between the tibia fixation and foot plate or mid-foot ring was performed with threaded rods, hinges or adjustable struts. The cancellous bone of the lateral malleolus was excised, fragmented into small pieces then placed between the distal tibia and the talus. The foot frame was then connected to the leg frame and compression was applied at the fusion site.

Post-operatively, the patients with Ilizarov fixator were allowed weight-bearing with ambulation as tolerated in most of cases. Patients with associated other fractures and those who were not medically stable were kept non-weight-bearing. Pin care began on the second post-operative day and was performed once daily after that. The patients were discharged after an average of two days and followed-up every two weeks for the first month and monthly subsequently.

At every visit, the patients were examined clinically for wound healing. The neurovascular status of the limb was also assessed as was any evidence of pin tract infection, postoperative ambulation status, need for walking aid, postoperative complications, union time and eradication of infection were recorded on every follow-up visit. They were also examined radiologically for bone healing and alignment at the ankle region. Union was defined as complete cortical bridging or bridging callus or trabeculae across the ankle joint and loss of lucency between fusion surfaces, absence of pain and motion when stress was applied to the ankle joint during post-operative clinical examination.

## Results

**Fig. 2:**
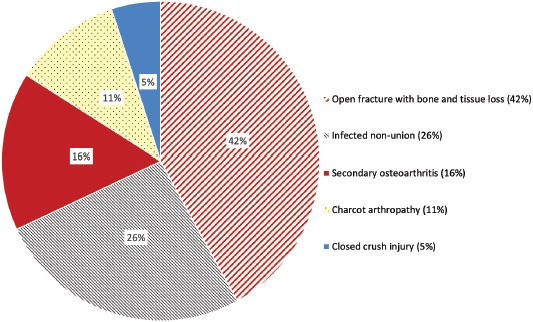
Indications for ankle arthrodesis.

**Fig. 3:**
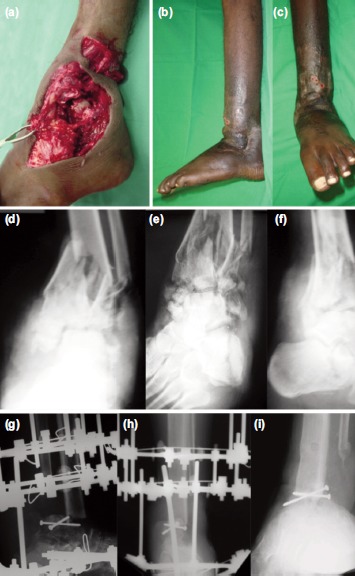
(a) Intra-operative photographs showing comminuted fracture left ankle. (b,c) Photographs after ankle fusion and removal of Ilizarov. (d,e) Pre-operative radiographs anteroposterior, mortis and lateral views showing comminuted fracture left ankle. (f,g) Immediate postoperative radiographs showing ankle arthrodesis with Ilizarov anteroposterior, lateral views. (h,i) Fifteen months post-operative radiographs showing fusion Ilizarov anteroposterior, lateral views.

The mean age of the patients was 45 (+/-11.5) years, and 13 (67%) were male and six (33%) were female. Fusion was successful in all 19 patients (100%). Radiological union took a range from 6-12 weeks, mean time to union was 8 weeks. In one outlier case, in a morbidly obese patient with multiple co-morbidities including hypertension, diabetes, ischemic heart disease, hyperlipidemia, asthma and on antituberculosis therapy and with bilateral foot drop due to a previous spine surgery, union took 81 weeks. Road traffic accidents, falls and earthquake were the three mechanisms of injury identified. The left lower limb was the most frequent site involved (50%). The most frequent indication for ankle arthrodesis was severe open fractures with soft tissue (Fig. 2) and bone loss eight patients (42%) (Fig. 3), infected nonunion six patients (26%) followed by Charcot arthropathy two patients (11%), osteoarthritis, three patients (16%) and the least frequent indication was crush injury one patient (5%). The average hospital stay was two days. When evaluated, seven of participants (35%) were found to have previous fixation for similar injuries, in addition 16 patients (83%) had positive history of previous surgeries due to other non-relevant reasons. The mean operative time was 2.5 (+/-1) hour.

Immediate post-operative ambulation status was full weight bearing (FWB) in 16 patients (83%) of the patients while three patients (17%) had non-weight bearing (NWB) due to associated injuries and co-morbidities. Post-procedure, 11 patients (67%) of the patients required some form of support for walking for initial 2-3 weeks. Post-operatively, three patients had pin tract infection requiring intravenous antibiotics. Only one participant required bone grafting due to bone loss from previous surgery. The range of follow-up period was 10-45 months. External Fixator Index averaged 45-450 days. The Ilizarov application was kept in place for 450 days (15 months) in a severe deformity and mal-union of right femur and tibia and foot of 18-year old neglected fractures in a 30-year old female.

## Discussion

This study retrospectively analysed the outcome and complication in 19 patients, with 20 ankles treated with arthrodesis by the llizarov technique. Ankle arthrodesis with Ilizarov was associated with higher union rate and shorter time to union, eradication of infection and deformity.

Advanced arthritis can be treated with internal fixation, external fixation or total ankle arthroplasty. Ankle arthrodesis with Ilizarov is associated with less damage to soft tissue, periosteum and vascularity than internal fixation techniques, thereby making it an ideal method of management in patients with soft tissue compromise and patients with peripheral vascular disease, diabetes mellitus, and Charcot arthropathy^12, 13^ which otherwise would end in amputation^14^. Ilizarov fixator has been regarded as a last resort for limb salvage in these cases^15^. The greater union rate in the Ilizarov technique might be related to the stability of the ring fixator and its ability to produce compression at the fusion site and stimulating bone healing^16^. Ilizarov technique addresses all three objectives of any joint arthrodesis which are successful union without deformity, stability during the fusion process through compression across the joint and elimination of instability^17^.

For a patient with extensive trauma at the ankle; ankle arthrodesis with Ilizarov has the advantages of allowing early weight bearing and has the potential to permit adjustment for correction of hindfoot alignment. Using Ilizarov bone transport technique, segmental bone loss at ankle may be reconstructed and is a potentially limb salvageable technique in complex ankle fracture^18^. The ankle joint cannot withhold deformity or articular incongruity after trauma. Studies have shown that this leads to pain and progressive ankle arthrosis^1^.

Post-traumatic osteoarthritis occurs following a variety of joint injuries, most commonly and predictably following injuries that disrupt the articular surfaces, leading to a mechanical insult to the cartilage matrix that affects chondrocyte function, attributed mainly to the initial joint injury and to elevated cartilage stresses from residual surface incongruity^19, 20^.

Total ankle replacement has certain theoretical advantages over ankle arthrodesis^19^. Gait is affected less, and adverse effects on the adjacent joints are not expected^19^. The Ilizarov technique can be an alternative salvage method in such cases. Salem *et al* reported on a group of 22 patients treated with the Ilizarov technique for posttraumatic ankle arthritis complicated by infection^21^.

Charcot neuroarthropathy had been a major indication for fusion. Charcot arthropathy is joint destructive process that leads to ankle instability, foot deformity, infection and amputation. The aim of treatment is to restore alignment and stability and achieve a plantigrade foot that is free of ulcers^16^. Arthrodesis can achieve these goals but surgical arthrodesis in Charcot neuroarthropathy has a high failure rate^16^. Because of infection, softening of bone and bone resorption, open reduction and internal fixation is associated with high complication rate. Ilizarov fixator is a versatile device that has the ability to correct the deformity gradually in the postoperative period with minimal disruption of soft tissue and maintain stable construct even in the presence of soft bone^22^. Charcot neuroarthropathy was of fourth most importance in our subjects. In our part of the world, patients suffer more from road traffic accidents and trauma which were the most common indications for ankle fusion in our study.

The union rate in our study was 100%. The union rate in the literature vary from 78% reported by Salem *et al*^21^ to 100% by Kawoosa *et al*^28^, and Li J *et al*^30^ (Table I).

**Table I:** Comparison of our current study with literature for number of patients, ankle fusion rate, complications, time wearing frame and fusion time

Study	Number of patients	Ankle fusion rate (%)	Complications (number of patients)	Time wearing frame (months)	Fusion time (weeks)
Current study	19	100	Pin tract infection (3)	7	6-12
	8 (Open fracture)		Non-union (0)		
	5 (Infection)				
	3 (Secondary osteoarthritis)				
	2 (Charcot arthropathy)				
	1 (Others)				
Li *et al*^30^ (2017)	31	100	Infection (1)		
	19 (Traumatic arthritis)		Non-union (0)		
	6 (Osteoarthritis)		Mid foot pain (3)		
	4 (Rheumatoid arthritis)				
	2 (Other)				
Kawoosa *et al*^28^ (2015)	16	100	Pin tract (5)		14
	7 (Post traumatic arthritis)				
	3 (Septic arthritis)				
	4 (Failed arthrodesis)				
	1 (Ankle instability)				
	1 (Rheumatoid arthritis)				
Fragomen *et al*^27^ (2012)	91	84	Non-union (15)	6.5	
			Broken fixation (3)		
			Severe deep infection (1)		
			Cellulitis (3)		
Karapinar *et al*^29^ (2009)	11	90			
	11 (Charcot neuroarthropathy)				
Fabrin *et al*^23^ (2007)	12	50			
	12 (Charcot neuroarthropathy)				
Salem *et al*^21^ (2006)	18	78			
	Infection				

Potential complications in the short term include nonunion, malalignment, and deep infection^21, 24^. The most common complication in our study was pin tract infection 15% which vary from 10% to 20% in the literature^17^. We report no nonunion or malunion or severe infection. Disturbed gait and adjacent joint arthritis are also described as substantial risks after fusion^25^, this was contrary to our findings. Total ankle replacement can potentially overcome these disadvantages, but the rate of subsequent major complications is reportedly higher than after arthrodesis^26^.

The use of the Ilizarov frame provides a successful salvage method that offers solid bony fusion, optimal leg length, and eradication of infection in complex ankle pathology or failed previous arthrodesis, and this is one of the strengths of our study.

The limitations of this study are the small sample size and a single center retrospective analysis. Clearly, a large sample size with randomisation should be done to relate our results to the defined population.

## Conclusion

The Ilizarov technique has a high union rate and leads to general improvements in clinical outcome. It may be considered for any ankle arthrodesis but is especially useful in complex cases such as revisions, soft-tissue compromise, infection and in patients at risk for non-union and should be considered a primary option where expertise is available. Early mobilisation and weight bearing are added benefits.

## Conflict of Interest

The authors declare no conflict of interest or external funding for this project.
